# Altered expression of miRNAs in a dihydrotestosterone-induced rat PCOS model

**DOI:** 10.1186/1757-2215-6-36

**Published:** 2013-05-15

**Authors:** Md Munir Hossain, Mingju Cao, Qi Wang, Ji Young Kim, Karl Schellander, Dawit Tesfaye, Benjamin K Tsang

**Affiliations:** 1 Institute of Animal science, Animal Breeding and Husbandry Group, University of Bonn, Endenicher allee 15, Bonn 53115, Germany; 2 Department of Animal Breeding and Genetics, Bangladesh Agricultural University, Mymensingh 2202, Bangladesh; 3 Reproductive Biology Unit and Division of Reproductive Medicine, Department of Obstetrics & Gynecology and Cellular & Molecular Medicine, University of Ottawa; Chronic Disease Program, Ottawa Hospital Research Institute, The Ottawa Hospital (General Campus), Ottawa, ON K1H 8L6, Canada; 4 World Class University Major in Biomodulation, Department of Agricultural Biotechnology, College of Agriculture and Life Sciences, Seoul National University, Seoul 151-921, Republic of Korea; 5 Ottawa Hospital Research Institute, The Ottawa Hospital (General Campus), 501 Smyth Road, Mail Box #511, Ottawa, ON K1H 8L6, Canada

**Keywords:** PCOS, miRNA, Dihydrotestosterone

## Abstract

**Background:**

The polycystic ovary syndrome (PCOS) is a complex and heterogeneous endocrine condition characterized by hyperandrogenism, hyperinsulinemia, insulin resistance and chronic anovulation. Regulation and interaction of a multitude of genes required for follicular development are found to be altered in PCOS. MicroRNAs (miRNAs) mediate posttranscriptional gene regulation by binding to the 3´ untranslated region of mRNAs to either inhibit or enhance translation. However, the extent and regulation of miRNA expression in PCOS is poorly understood and the current study is the first to describe altered expression of miRNAs in PCOS.

**Methods:**

A chronically androgenized [5α-dihydrotestosterone (DHT)-treated] rat model which recapitulates many of the phenotypes of human PCOS, and miRNA PCR array was used to investigate the expression of 349 miRNAs in DHT treated rat ovaries. The ovarian expression of several selected miRNAs was also analyzed by in situ localization experiment.

**Results:**

DHT-treated rats exhibit increased body weight, disrupted estrus cyclicity, decreased insulin sensitivity and decreased ovarian weight, with the latter phenomenon readily rescued by gonadotropin treatment in vivo. In general, 24% of the 349 miRNAs investigated were found to be differentially expressed between DHT-treated and control rats. Most of the differentially expressed miRNAs were found to be predominantly localized in the theca cells of the follicles. In silico analysis of the potential target genes of dysregulated miRNAs revealed their possible involvement in various pathways in the regulation of ovarian function.

**Conclusion:**

Our current findings suggest that miRNAs are differentially regulated in hyperandrogenism, a condition possibly involved in the dysregulation of steroid hormone receptors and intra-ovarian factors, and that miRNAs may be involved in the etiology of PCOS.

## Introduction

Polycystic ovary syndrome (PCOS), a multi-factorial endocrine condition, affects up to 5-10% of women in reproductive age and accounts for 75% of anovulatory infertility [1-3]. PCOS is a heterogeneous syndrome with complex pathologies with follicle growth arrest at the small antral stage, minimal granulosa cell proliferation, hyperthecosis and hyperandrogenemia, and chronic anovulation [1]. It is associated not only with infertility, but also with increased risk of metabolic disorders, such as insulin resistance, diabetes, obesity, and cardiovascular diseases [4]. Both genetic and environmental factors are known to contribute to the pathogenesis of PCOS; however, its etiology is unclear.

Differential gene expression is evidenced in human PCOS ovaries when compared to the normal ones [5]. Genomic studies in PCOS have revealed a variety of altered genes that fall into many functional categories, including cell division, apoptosis and regulation of gene expression and metabolism. While genes involved in steroidogenesis, including those related to retinoic acid biosynthesis and luteinizing hormone-responsive gene, are generally up-regulated in theca cells of PCOS subjects [6], those associated with Wnt signalling and ovarian folliculogenesis appear to be down-regulated [7,8]. Although ovarian apoptotic genes are altered in PCOS, the net effect of these changes is unclear [7]. In addition, significant differences in mRNA abundance in oocytes from PCOS patients have also been reported and cluster analysis of 374 genes indicated an association with chromosomal alignment and segregation in mitosis and/or meiosis [9].

Despite ample evidences on gene dysregulation in PCOS ovary as reported above, precisely how these genes are transcriptional and post-transcriptional regulated is poorly understood. New advances in post-transcriptional gene regulation have resulted from the discovery of hundreds of miRNAs in different mammalian species. Diverse expression pattern of miRNAs and the high abundance of potential targets in the ovary have led to the notion of their importance in the regulation of ovarian function. A large number of miRNAs has now been identified in the ovary [10,11] and found to be regulated by gonadotropins during follicular development [12,13]. Recent studies indicate that miRNAs are involved in the regulation of steriodogenesis, cell proliferation and apoptosis in human granulosa cells [14,15]. Differential expression of miR-23a, miR-23b,miR-542–3p, miR-211, and miR-17–5p in granulosa/cumulus cells from women undergoing assisted reproduction suggests aberrant miRNA expression may be an underlying etiology in female infertility [16,17]. Furthermore, miRNAs have been implicated to play a role in obesity and metabolic syndrome, including insulin resistance [18], conditions often associated with PCOS. However, whether miRNAs contribute to the follicle arrest, dysregulated steroid production and insulin resistance in PCOS is not known. Understanding how miRNAs are regulated in the ovary and the identification of their specific targets and functions may offer novel insights into the etiology of PCOS and the development of target-specific gene regulation for its treatment.

Hyperandrogenism is one key feature in human PCOS patients and circulating levels of androgens, such as testosterone, androstenedione and dihydrotestosterone (DHT), are elevated in these patients [19,20]. A number of animal models have been developed that allow one to focus on different aspects of PCOS pathology, including ovarian follicular and metabolic features [21-23]. A recent report on a rat PCOS model involving chronic DHT implant mimics the hyperandrogenic status and exhibits both polycystic ovaries and metabolic abnormalities similar to those in human PCOS [23], allowing one to examine not only ovarian characteristics but also metabolic features of PCOS.

We hypothesize that PCOS is associated with dysregulation of ovarian miRNAs which may be involved in post-transcriptional regulation of genes involved in the ovarian pathology. Taking advantage of DHT-induced rat PCOS model, we tested this hypothesis by comparing the expression of 349 miRNAs between DHT-treated vs untreated rat ovaries. Moreover, selected candidate miRNAs were investigated for their localization in follicular cells. Results of the present study revealed that PCOS is associated with differential expression of regulatory non-coding miRNAs in rat ovaries.

## Materials and methods

### DHT-filled silastic capsules preparation

The DHT-filled capsules were prepared as previously described [24]. Silastic tubing (I.D. 1.98 mm x O.D. 3.18 mm; Dow Corning, Cat. No. 2415577, Midland, MI), cut to an appropriate length to achieve a desired surface area of 299.70 mm^2^, was filled with DHT (Sigma) from 1 ml syringe. The tubing was closed at each end (3 mm) with a sealant (Silicone Type A, Dow Corning Cat. No. 891, Midland, MI), ensuring that adhesive is in contact with tubing walls and that there are no air bubbles. Control capsules were empty with only sealant on both ends. After overnight dry, capsules were rinsed for 2 days in 3% bovine serum albumin (BSA) in phosphate buffer saline (PBS) with 0.1% NaN_3_ solution, thoroughly washed with PBS and sterilized by dipping briefly in 70% ethanol before use.

### Animal preparation

Sprague Dawley rats (Charles River, Montreal, Canada) were maintained on 12-h light, 12-h dark cycles and given food and water ad libitum. All procedures were carried out in accordance with the Guidelines for the Care and Use of Laboratory Animals, Canadian Council on Animal Care, and were approved by University of Ottawa and Ottawa Hospital Research Institute Animal Care Committee.

### Animal surgery, DHT capsule implantation and animal care

Thirty-six female rats at 21 days of age were randomly divided into two groups (control vs. DHT group) and were implanted with a DHT-filled silicone capsule continuous-releasing (83 μg per day; empty capsule for controls) for 12 weeks to mimic the hyperandrogenic state in women with PCOS, whose plasma DHT levels are approximately 1.7-fold higher than those of healthy control [23]. Animals were weighed weekly to monitor body weight gain and euthanized at 12 wks post-implantation. Ovaries were collected for analyses.

### Determination of estrous cycle phases of rat

The estrous cycle of rats was monitored by microscopic analysis of vaginal smear obtained every morning for 12 days from 10–11 wks post-implantation. Predominant cells associated with each phase of the estrus cycle were: epithelial cells (proestrous; P), enucleated cornified cells (estrous; E), equal mix of leukocytes and cornified epithelial cells (metestrous; M) and leukocytes (diestrous; D) [25].

### Determination of insulin sensitivity

Insulin sensitivity test (IST) was performed after 12 h overnight fasting at 10 wks of surgery. Human insulin (0.2 U/100 g body weight intravenously, Novo Nordisk Canada Inc. Mississauga, ON, Canada) was administered to control or DHT-treated rats through tail vein and blood samples were obtained from the tail at 0, 5, 10, 20, 40, 80, 160 min. after insulin injection. Glucose levels were determined by glucose test strips (ACCU-CHEK, Roche). The insulin sensitivity index *K*_*ITT*_ was calculated from the slope of a linear portion of the regression line of natural logarithm of glucose versus time. Raw data were transformed by Log *K*_*ITT*_ for analysis. *K*_*ITT*_ = (0.693/t_1/2_) × 100, t_1/2_ represents the half-life of glucose decay after insulin injection [26].

### Ovarian tissue collection and morphological evaluation

Pairs of ovaries were excised, and weighed. For morphological evaluation, whole ovaries were embedded in paraffin block and longitudinal sections (4 μm thick) were stained with hematoxylin and eosin. Since the considerable discrepancy in estimating follicle numbers exists between studies due to different section thickness and correction factors adopted [27], the percentage of number of cystic follicles (FC) and corpus lutea (CL) over total follicular structure (including all kinds of follicles and CL) per ovary were determined. A FC was defined as an collapsed anovulatory follicle exhibiting the absence of oocyte, reduced granulosa cell layer, but intact theca layer. The maximal longitudinal ovarian sections were investigated to count the numbers of FC and CL, mean of the percentage of FC and CL from 3–5 sections after sampling every fifth section were defined as the relative values per ovary from 5 individual animals. In an additional experiment, three rats from DHT group at each time point were administered with 20 IU of equine chorionic gonadotropin (eCG; Sigma; PBS as control) for designated time (0–30 h) to test their ovarian responsiveness to gonadotropin in vivo.

### miRNA expression profiling

Ovarian cortex tissues were snap-frozen in liquid nitrogen and stored at −80°C until analysis. Total RNAs including small RNAs from 3 independent DHT (DHT1-3) treated and 3 non-treated control (CTL1-3) ovaries have been isolated using miRNeasy mini kit (Qiagen, Hilden, Germany) following manufacturer’s protocol. Immediately after isolation, contaminating DNA and divalent cations from the RNA samples were removed using TURBO DNA-free™ kit (Ambion, Foster City, CA) according to instruction of the manufacturer. The quality and the concentration of the RNA samples were assessed by NanoDrop 8000 spectrophotometer (NanoDrop, Wilmington, Delaware, USA). Subsequently, a total of 250 ng purified RNA from each samples were reverse transcribed for qPCR analysis, using the QuantiMir™ RT systems (System Biosciences, Mountain View, CA).

MiRNA expression analysis was performed using miRNA PCR array platform containing 349 well characterized Rat miRNAs including 3 endogenous controls, namely Rat U6 snRNA, RNU43 snoRNA & U1 snRNA (System Biosciences, Mountain View, CA, Cat # RA680A-1) according to the user manual of miRNome microRNA Profilers QuantiMir™ (System Biosciences, Mountain View, CA) with some modifications. Briefly, 20 μl of cDNA from each sample mixed with 1300 μl of Maxima 2x SYBR Green, (Fermentas Gmbh, Leon-Rot, Germany, Cat # K0222), 50 μl of universal reverse primer and 800 μl of nuclease-free water. Five microlitres of mixed cDNA cocktail were dispersed into each well of 384 well PCR plate containing 1 μl of pre-aliquoted miRNA specific primer. Multichannel laboratory automation workstation was used for pipetting the mix and mixing the reaction into 384 well plates (Biomek® NX^P^, Beckman Coulter, Krefeld, Germany). The assays were performed in ABI 7900 HT real time PCR system (Applied Biosystems, Foster City, CA, USA) with thermal cycling programme at 52°C for 2 min, 95°C for 10 min, 40 cycles of (95°C for 15 sec, 60°C for 1 min). Dissociation stage was included at the end of the programme to assess the Tm of the PCR amplicons and to verify the specificity of the amplification reactions. In addition, amplification was verified for randomly selected reactions through 2.5% agarose gel electrophoresis. Data were analysed by ΔΔC_t_ method and normalized by the geometric mean of the three endogenous controls through System Bioscience’s free Software developed to analyse PCR Array data for Rat miRNome (System Biosciences, Mountain View, CA), equipped with t test. A fold regulation of ≥ 2. P ≤ 0.05 was considered as significant in expression differences.

### Localization of selected miRNAs in ovarian cryo-section

Ovaries were fixed in 4% paraformaldehyde (pH 7.3; 4°C, overnight) followed by incubation in 0.5M Sucrose in DEPC-treated 1X PBS (4°C, overnight). They were embedded in Tissue-Tek Oct reagent (Shandon Cryomatrix, Thermo Cat# 6769006) in a tissue path base mold, underwent a slow freezing process (−1°C/min rate) in a freezing container (NALGENE™ Cryo, Cat# 5100–0001). Serial ovarian sections (10 μm) were cut at −23°C using rapid sectioning cryostat (Leica microsystem Nussloch GmbH, Heidelberger, Germany) and mounted on SuperFrost® Plus slides (Menzel-Glaeser, Germany) at −80°C until hybridization began. Sections were checked for quality and required cell content at a regular interval by staining with TB in a light microscope. Post-fixation, acetylation and proteinase K treatment was done as described previously [28]. Two hours of pre-hybridization was performed at 52°C in hybridization solution (50% formamide, 5x sodium chloride/sodium citrate [SSC; pH 6.0], 0.1% Tween-20, 50 μg/ml heparin, and 500 mg/ml yeast tRNA). Ovarian sections were incubated overnight at 52°C in a hybridization buffer containing 3'-Digoxigenin (DIG) labeled LNA-modified oligonucleotide probes (1pM) for miRNA, U6 RNA and scrambled miRNA (Exiqon, Vedbaek, Denmark) in a humidified chamber. Blocking, incubation with anti-DIG-AP antibody, washing and color development with alkaline phosphatase substrates solution [5-Bromo-4-chloro-3-indolyl phosphate/Nitro blue tetrazolium (BCIP/NBT)] were performed as described previously [28]. Vector® Methyl Green (Vector laboratories, Burlingame, CA) was used as nuclear counter-stain.

### Statistical analyses

All data were analyzed using Prism 5.0 statistical software (GraphPad, San Diego, CA). The results were presented as means ± SEM as detailed in figure legends. Differences in body weight gains, follicle cysts and corpus luteum percentage were analyzed by two-way ANOVA, ovarian weight between groups were analyzed by one-way ANOVA and followed by Bonferroni post-hoc test. Insulin sensitivity in PCOS and control groups was compared by Student t-test. Statistical significant difference was defined at P < 0.05.

## Results

### Characterisation of PCOS rat model

#### Body weight increased in PCOS rats

**Figure 1:**
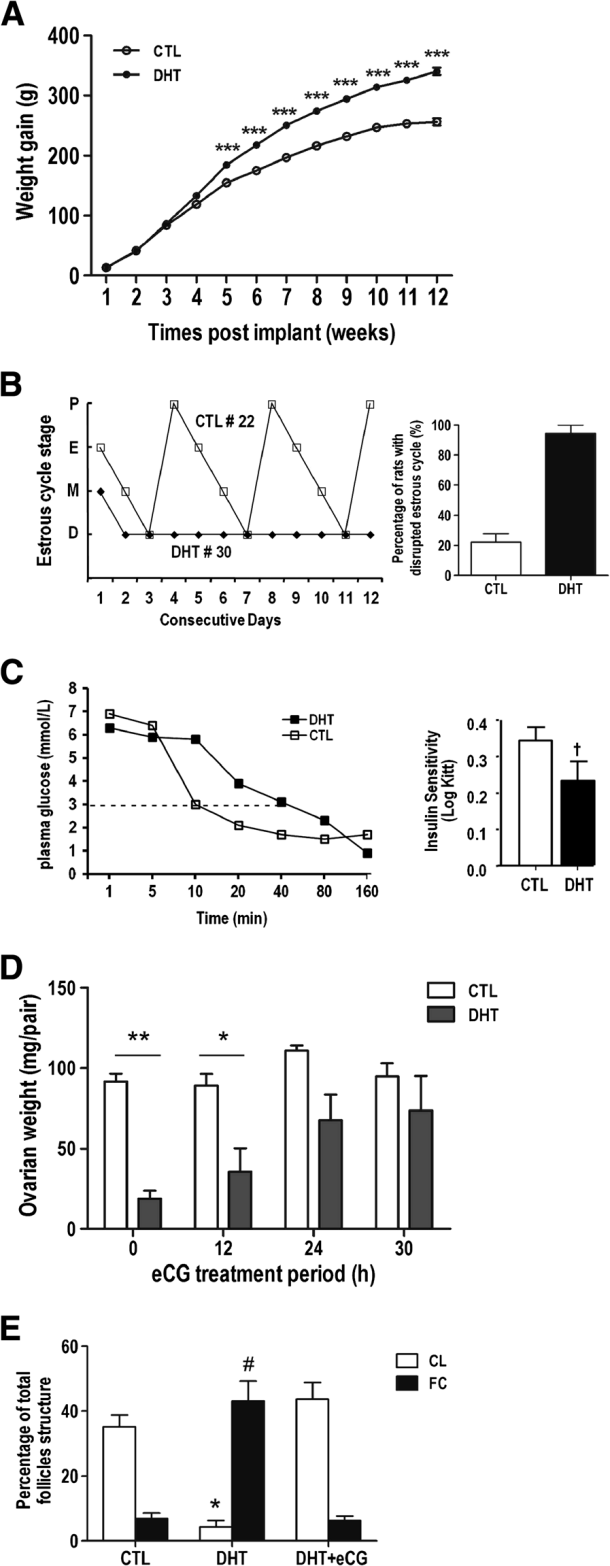
**DHT-treated rats show higher body weight gain, disrupted estrous cycles, insulin resistance, responsiveness to gonadotropin and ovarian features.** (**A**) Weight gain of DHT-treated rats are significantly higher than CTL animals after 5 weeks DHT implant (***, P < 0.001). (**B**) Estrous cycle was evaluated by microscopic analysis of the predominant cell type in vaginal smears obtained daily from 10–11 weeks post-implantation. Estrous cycle patterns were shown in two representative rats from each group. P: proestrous; E: estrous; M: metestrous; D: diestrous. 94% of DHT-treated rats show irregular estrous cycle versus 22% in CTL rats. (**C**) Insulin Sensitivity Test (IST): Animals were injected with insulin (0.2U/100g Body weight) through tail vein and plasma glucose levels were measured by glucose meter at the time indicated. Data was transformed by Log *K*_ITT_ for analysis. *K*_ITT_=0.693/t_1/2_x100, t_1/2_ represents the half-life of glucose decay after insulin injection (†P < 0.05). (**D**) 20 IU eCG was injected to DHT-treated rats for designated time (0–30 h) and ovarian weight was assessed. Reduced ovarian weight in DHT-treated rats was reversed by eCG treatment *in vivo* (*, P <0.05; ** P < 0.01). (**E**) Corpus luteum and cystic follicle numbers were counted in the maximal longitudinal ovarian sections and percentage of FC or CL over the total follicle structure per section were presented. Data showed that PCOS rats was bearing large number of cystic follicles accompanying with anovulation (CL percentage in control vs DHT was 35%, 4%, *P < 0.05; cystic follicles (FC) was 7% vs 43%, respectively, # P < 0.05).

Body weight for individual animal was weighed weekly. As shown in Figure 1A, weight gain in DHT-treated rats was significantly higher than control animals over the experimental period (340.32 ± 20.66 vs. 256.49 ± 21.31g, N = 18, P < 0.001), beginning at 5 weeks after implantation.

#### Irregularity in estrous cycle in DHT-treated rat

Estrous cycle was evaluated by microscopic analysis of the predominant cell type in vaginal smears obtained daily from 10–11 weeks post-implantation. Estrous cycle pattern in a representative rat from each group is shown (Figure 1B). During 12 days of evaluation, control rat # 22 had 3 regular estrous cycles, whereas DHT-treated rat # 30 started at metestrous and then showed prolong diestrous phase. 94% of animals in DHT group shown disrupted estrous cycle compared to 22% of animals in control group (Figure 1B, P < 0.001, N = 17).

#### DHT-treated rats showed insulin resistance

In order to examine the metabolic phenotype of rats which received DHT, insulin sensitivity test was performed. Rat plasma glucose levels were determined by glucose test strips after insulin challenge. The insulin sensitivity index *K*_*ITT*_ was calculated and a lower *K*_*ITT*_ suggests decreased insulin sensitivity or insulin resistance [26]. Our data shows reduced insulin sensitivity in DHT-treated rats compared to control (Control rats Log *K*_*ITT*_ = 0.34 ± 0.03 vs. DHT-treated rats 0.23 ± 0.05, N = 17, P < 0.05) (Figure 1C). As indicated as dotted line in Figure 1C, it took 10 minutes for the Control rat # 12 to change the basal glucose level from 6.9 mmol/L to 3 mmol/L, whereas it took 40 minutes for the glucose levels of DHT-treated rat # 5 to decrease from 6.3 to 3 mmol/L. A glucose curve shift to the right was evident in DHT-treated rats when compared to control animals.

#### Ovary weights were lower in DHT-treated rats

Pair ovary weight in DHT-treated rats was significantly lower than their control counterparts (0 h: 18.93 ± 4.85 mg vs. 91.90 ± 4.48 mg. P < 0.001) (Figure 1D). To test if DHT-treated ovaries are responsive to gonadotropin, three DHT-treated rats per group were administered with 20 IU of eCG (PBS as control) for designated time (0–30 h) and the ovaries were weighed in an additional experiment. Interestingly, the reduction of ovary weight in DHT-treated rats was markedly rescued with longer eCG treatment, indicating that this PCOS phenotype was responsive to gonadotropin (Figure 1D).

#### DHT-treated rats exhibited anovulation

To assess ovarian feature of PCOS rat, ovarian morphology in sections from CTL or DHT-treated rats was examined by H&E staining. Percentage of cystic follicle (FC) and corpus luteum (CL) over the total follicular structure were estimated in the longitudinal ovarian sections, as described in Materials and Methods. Estimation of relative value of FC and CL number, regardless of the total follicle numbers and their stages, is sufficient to evaluate disruption of ovarian follicular development as seen in PCOS. In control rat, CL accounted for 35% of total follicular structure per section, whereas those in DHT-treated rats were 4.3% (Figure 1E, P < 0.05). In contrast, follicle cysts in DHT-treated rats accounted for 43% of total follicle structure, which was significantly higher than in control rat (7%) (P < 0.05). Gonadotropin treatment reactivated follicular development in the cystic ovaries and induced ovulation, as the presence of CL increased to 43.6% in eCG-treated rats, while FC percentage declined to 6.3% over the total follicle structure (Figure 1E).

### Differential expression of miRNAs in the rat PCOS

**Figure 2:**
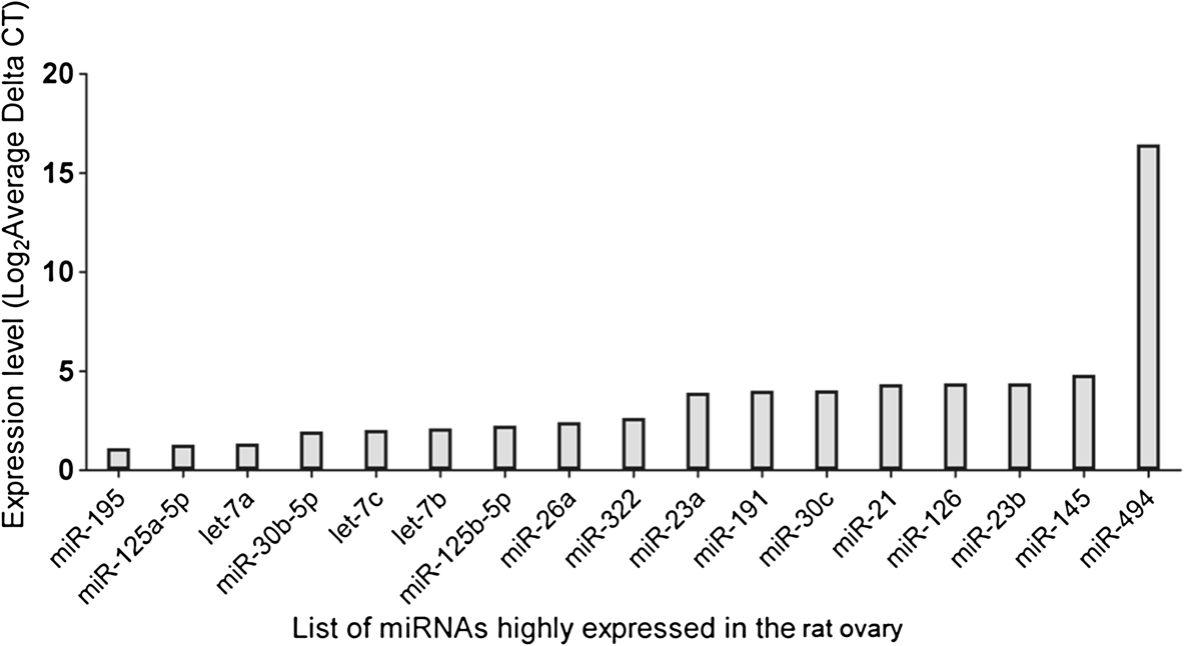
MiRNAs predominantly expressed in normal rat ovary compared to the expression level of other miRNAs.

Among the 346 miRNAs analysed, 25 miRNAs were found to be highly expressed in the normal ovary when compared to the expression level of other miRNAs (Figure 2). Most of these 25 miRNAs were also found to be highly expressed in DHT-treated ovary, although the extent of their expression was different compared to the normal ovary. Thus, the highly expressed miRNAs in control or DHT-treated ovary may be designated as ovarian miRNAs. These include rno-miR-195, rno-miR-125a-5p, rno-let-7a, rno-miR-16, rno-miR-30b-5p, rno-let-7c, rno-let-7b, rno-miR-125b-5p, rno-miR-221, rno-miR-222, rno-miR-26a, rno-miR-322, rno-miR-23a, rno-miR-191, rno-miR-30 family, rno-miR-21, rno-miR-126, rno-miR-23b, rno-miR-145 and rno-miR-494.

**Figure 3:**
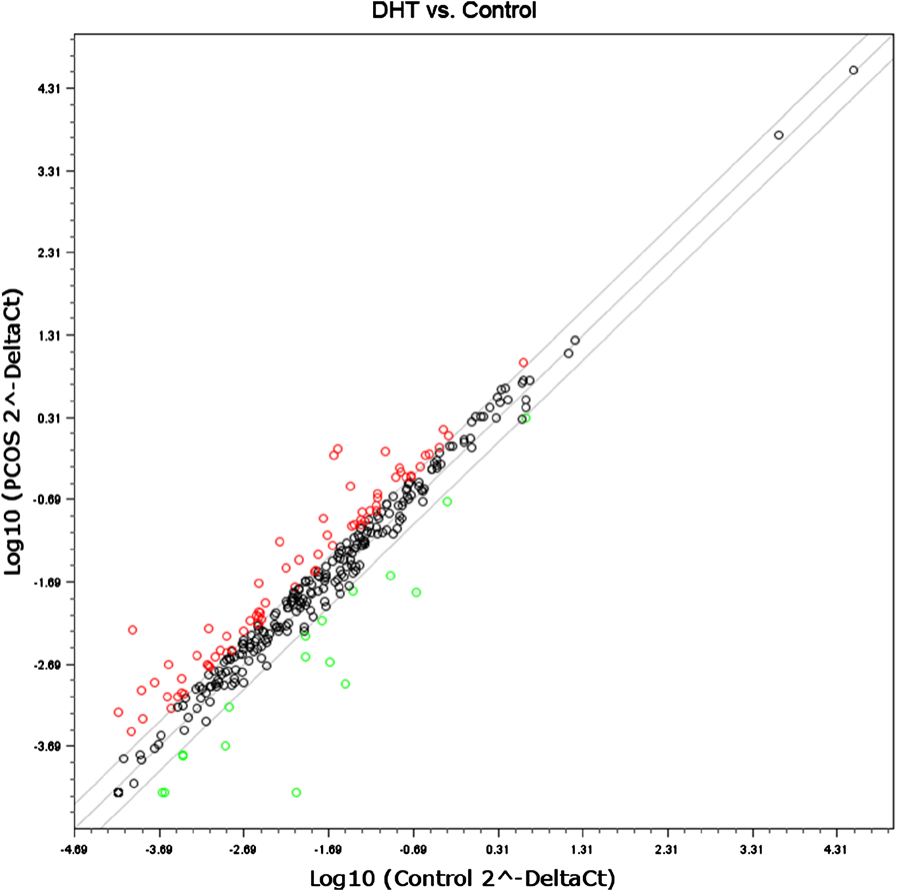
**Scatter plot of miRNA expression by miRNA microarray analysis. The expression profile of 346 miRNAs as log10 (2^-Delta Ct) in PCOS (DHT) and in control group (CTL) is plotted.** Specific miRNA expression positioned on the mid-line indicates no differential expression. Whereas lines above or under the mid line denotes the boundary of 2 fold regulation of miRNA expression. Red and green circles represent the number of miRNAs in DHT ovary significantly (P < 0.05 and 2 fold-change cut off) over-expressed and down-regulated, respectively.

**Figure 4:**
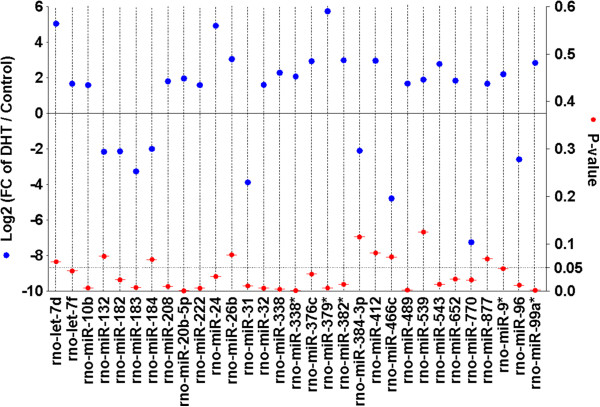
**Plot diagram of the magnitude of fold regulation of most differentially regulated miRNAs in DHT ovary compared to control.** The degree of expression difference by 2 or more fold regulation (log2 of Fold change of DHT/control: left Y axis) is presented by blue circle with their corresponding P-value (red circle on the right Y axis) of 31 screened miRNAs in DHT ovary compared to control.

Expression of 346 miRNAs in ovaries of DHT-treated and control rats were compared and a fold change of 2 or more and with the P values equal to or less than 0.05 were considered statistically significant. In general, 24% miRNAs were differentially expressed between DHT and CTR ovary. Among the differentially expressed miRNAs, most of the miRNAs (80%) were evidenced as over-expressed in DHT ovary. A total of 17 and 72 miRNAs were found to be down- and up-regulated, respectively in DHT compared to the CTL ovary (Figure 3). A list of differentially expressed miRNAs (Fold change ≥ 2 and their corresponding P value) is presented in Figure 4. Beside this group, miRNAs which were also highly abundant in DHT-treated ovaries are rno-miR-221, rno-miR-222, rno-miR-25, rno-miR-26b, rno-miR-379*, rno-let-7d, rno-miR-24, rno-miR-673, rno-miR-26b, rno-miR-335, rno-miR-382*, rno-miR-412, rno-miR-99a*, rno-miR-543, rno-miR-674-3p, rno-miR-409-3p. MiRNAs found to be primarily down-regulated in DHT-treated rats includes rno-miR-770, rno-miR-466c, rno-miR-21, rno-miR-31, rno-miR-182, rno-miR-183, rno-miR-96, rno-miR-132, rno-miR-182, rno-miR-384-3p and rno-miR-184.

### In situ localization of differentially expressed miRNAs in rat PCOS

**Figure 5:**
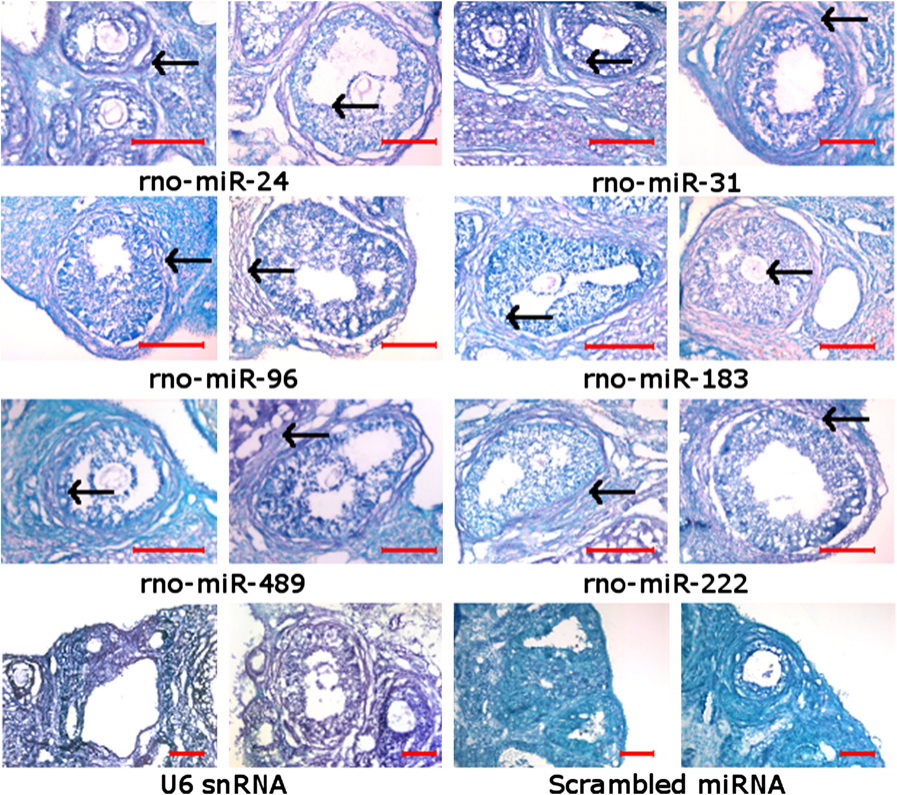
***In situ *localization of miRNAs across different follicular stages in DHT-treated ovaries.** Expression of miRNAs is detected as purplish blue color precipitate of BCIP/NBT from the hybridization of specific miRNA probe, whereas green color indicates cellular DNA (stained by methyl green). Scale bar indicates 100 μm and black arrow shows detected area for the particular miRNA.

Cell-specific expression of differentially regulated miRNAs across different follicular stages in control and DHT-treated rats were compared by in situ localization (Figure 5). These included rno-miR-24, rno-miR-31, rno-miR-96, rno-miR-183, rno-miR-222, rno-miR-489, U6 snRNA (positive control) and scrambled miRNA (negative control). Most of these miRNAs were localized in theca and granulosa cells as well as in oocytes at early stages of follicular development. Interestingly, miRNAs in the ovary undergoing or became cystic condition were differentially localized in the follicular cells in the advanced stages (Figure 5). Most of the miRNAs were localized in the follicular theca cells of DHT-treated ovaries. For example, rno-miR-96, rno-miR-31 and rno-miR-222 were exclusively expressed in the theca of cystic follicles. Whereas rno-miR-24 and rno-miR-183 were highly expressed in the theca and, to a lesser extent, in the granulosa cells of the cystic follicles (Figure 5), Rno-miR-31 and rno-miR-96 were present in the cumulus granulosa cells. Occasionally, expression of rno-miR-489 was most noticeable in the theca and cumulus cells of FC. Thus, most of the differentially expressed miRNAs in the cystic ovary were predominantly localized in the theca cells.

### Functional classification of differentially expressed miRNA

To explore and identify dysregulated molecular pathways resulting from altered miRNAs in DHT-treated ovaries, miRNAs highly enriched or markedly reduced in these ovaries were subjected to Ingenuity pathway analysis (IPA; http://www.ingenuity.com). MiRNA target genes and their respective molecular pathways were filtered and prioritized. Only highly predicted and experimentally validated targets were considered as the target genes of miRNA. Among the fourteen miRNAs mapped to the ingenuity databases, twelve (rno-let-7d, rno-miR-132, rno-miR-182, rno-miR-183, rno-miR-184, rno-miR-21, rno-miR-221, rno-miR-24, rno-miR-25, rno-miR-26b, rno-miR-31 and rno-miR-96) had 171 experimentally validated targets. When highly predicted genes were included to the outcome, a total of 2459 target genes were found to be targets of 14 differentially expressed miRNAs. The molecular pathways dysregulated by these differentially expressed miRNAs in DHT-treated ovaries was evaluated and included signalling by androgen, aldosterone, VEGF, FGF, growth hormone, 14-3-3, calcium, estrogen receptor, G-Protein coupled receptor, glucocorticoid receptor, and apoptosis as well as in ovarian cancer signalling and androgen and oestrogen metabolism (Table 1).

**Table 1:** Molecular pathways regulated by miRNAs in DHT-treated ovaries

***Molecular pathways***	***List of miRNA***
*Ovarian cancer signalling*	rno-miR-96↓, rno-miR-25↑, rno-miR-24↓, rno-miR-221↓, rno-miR-21↓, rno-miR-183↓, rno-miR-182↓, rno-miR-132↓, rno-let-7d↑
*Androgen signalling*	rno-let-7d↑, rno-miR-132↓, rno-miR-182↓, rno-miR-183↓, rno-miR-184↓, rno-miR-21↓, rno-miR-221↓, rno-miR-24↓, rno-miR-25↓, rno-miR-26b↑, rno-miR-31↓, rno-miR-335↑, rno-miR-384-3p↓
*Androgen & estrogen metabolism*	rno-miR-96↓, rno-let-7d↑, rno-miR-24↓, rno-miR-26b↑, rno-miR-21↓, rno-miR-26b↑, rno-miR-31↓
*Aldosterone signalling (epithelial cells)*	rno-miR-182↓, rno-miR-183↓, rno-let-7d↑, rno-miR-132↓, rno-miR-182↓, rno-miR-21↓, rno-miR-221↓, rno-miR-24↓, rno-miR-25↑, rno-miR-26b↑, rno-miR-335↑, rno-miR-384-3p↓, rno-miR-96↓
*VEGF signalling*	rno-miR-96↓, rno-miR-182↓, rno-miR-31↓
*RhoA signalling*	rno-miR-132↓, rno-miR-182v, rno-miR-183↓, rno-miR-384-3p↓, rno-miR-96↓
*14-3-3-mediated Signalling*	rno-miR-96↓, rno-miR-384-3p↓, rno-miR-335↑, rno-miR-31↓, rno-miR-26b↑, rno-miR-25↑, rno-miR-24↓, rno-miR-221↓, rno-miR-21↓, rno-miR-184↓, rno-miR-183↓, rno-miR-182↓, rno-miR-132↓, rno-let-7d↑
*Apoptosis signalling*	rno-miR-25↑, rno-let-7d↑, rno-miR-182↓, rno-miR-221↓, rno-miR-24↓, rno-miR-25↑, rno-miR-26b↑, rno-miR-31↓, rno-miR-335↑, rno-miR-384-3p↓, rno-miR-96↓
*Calcium signalling*	rno-miR-384-3p↓, rno-let-7d↓, rno-miR-132v, rno-miR-183↓, rno-miR-21↓, rno-miR-221↓, rno-miR-2↓5, rno-miR-26b↑, rno-miR-384-3p↓, rno-miR-96↓
*Estrogen receptor signalling*	rno-miR-182↓, rno-miR-96↓, rno-let-7d↑, rno-miR-132↓, rno-miR-182↓, rno-miR-24↓, rno-miR-25↑, rno-miR-26b↑, rno-miR-335↑, rno-miR-384-3p↓
*FGF Signalling*	rno-let-7d↑, rno-miR-132↓, rno-miR-182↓, rno-miR-183↓, rno-miR-21↓, rno-miR-221↓, rno-miR-384-3p↓, rno-miR-96
*G-Protein coupled receptor signalling*	rno-let-7d↑, rno-miR-182↓, rno-miR-183↓, rno-miR-21↓, rno-miR-24↓, rno-miR-25↑, rno-miR-26b↑, rno-miR-31↓, rno-miR-335↑, rno-miR-384-3p↓, rno-miR-96↓
*Focal adhesion kinase-1 (FAKn)*	rno-miR-25↑, rno-miR-384-3p↓, rno-miR-96↓, rno-miR-183↓, rno-miR-26b↑, rno-miR-182↓, rno-miR-21↓, rno-miR-24↓, rno-miR-31↓
*Glucocorticoid receptor Signalling*	rno-let-7d↑, rno-miR-182↓, rno-miR-221↓, rno-miR-24↓, rno-miR-25↑, rno-miR-26b↑
*Growth hormone Signalling*	rno-let-7d↑, rno-miR-182↓, rno-miR-183↓, rno-miR-21↓, rno-miR-24↓, rno-miR-25↑, rno-miR-26b↑
*Inositol phosphate metabolism*	rno-miR-335↑, rno-let-7d↑, rno-miR-132↓, rno-miR-182↓, rno-miR-221↓, rno-miR-24↓, rno-miR-25↑, rno-miR-26b↑, rno-miR-335↑, rno-miR-384-3p↓, rno-miR-96↓
*P53 signalling*	rno-miR-132↓, rno-miR-183↓, rno-miR-21↓, rno-miR-221↓, rno-miR-24↓, rno-miR-25↑, rno-miR-26b↑, rno-miR-31v↓, rno-miR-96↓

(↑ denotes upregulation and ↓ denotes downregulation).

## Discussion

PCOS is a complex and heterogeneous endocrine condition characterized by hyperandrogenemia, hyperinsulinemia, insulin resistance, and chronic anovulation. Using a rodent model for PCOS which recapitulates the reproductive and metabolic phenotypes of the human conditions [23], we have begun to examine molecular and cellular mechanisms underlying the dysregulated reproductive and metabolic functions of PCOS. We have demonstrated that chronically androgenised rats, exhibited increased body weight, disrupted estrus cyclicity, decreased insulin sensitivity and decreased ovarian weight. In the present study we have for the first time that these phenotypes are associated with dysregulation of gene regulatory noncoding miRNA.

Ovarian growth is the result of a series of complex and coordinated processes, which include morphological and functional changes in different follicular cells and their interactions. Among the small non-coding RNAs associated to various biological and physiological disorders (sncRNAs), miRNAs are the best characterized. Studies on the bovine and mouse ovary have demonstated the presence of miRNAs and suggested their potential role in ovarian functions [10,29]. Several intra-ovarian regulators and their receptors have been predicted as targets of this ovarian miRNAs, which mediate important intracellular events necessary for normal follicular development and functions.

Although miRNAs are expressed in the human, mouse and cow ovaries [10,14,29-34], little is known of their role and dysregulation in PCOS. The present study represents the first report on the dysregulation of miRNAs in a rat ovary chronically exposed to DHT, a condition mimicking the hyperandrogenic condition in human PCOS. Among the miRNAs examined, 79 miRNAs (24%) responded to the hyperandrogenic condition and interestingly, 80% of which were upregulated compared to the control group supporting the notion that hyperandrogenic condition down-regulates androgen receptors in the granulosa cells [35] which could be mediated by these upregulated miRNAs (rno-miR-379*, rno-let-7d, rno-miR-24, rno-miR-673, rno-miR-26b, rno-miR-335, rno-miR-382*, rno-miR-412, rno-miR-99a*, rno-miR-543, rno-miR-674-3p, rno-miR-409-3p). It has been reported that the expression of miR-143, let-7a, miR-15b is under negative control of follicle stimulating hormone (FSH) during follicular development [36] and may be involved in FSH-induced rat granulosa cell progesterone production [37]. Thus, it is possible that the down-regulation of miRNAs (rno-miR-770, rno-miR-466c, rno-miR-31, rno-miR-183, rno-miR-96, rno-miR-132, rno-miR-182, rno-miR-384-3p and rno-miR-184) observed in this study could be associated with promoted thecal hyperandrogenesis [37,38]. Similarly, stepwise artificial neural networks analysis revealed predictive miRNA signatures (miR-342, miR-299, miR-217, miR-190, miR-135b, miR-218) corresponding to oestrogen receptor status in breast cancer [39]. These findings raises the possibility that the modulated miRNA expression by exogenous DHT treatment activates estrogen receptors (ERs) to exert indirect steroidal actions on the follicular cells or directly on androgen receptor pathway for the regulation of androgen metabolism [40].

Recent evidences suggest that miRNAs are involved in the regulation of steroid hormone receptors (SHRs) in ovarian follicle growth and hormone-responsive cancers. Follicular development is the result of tightly regulated activities of FSH, estrogen, androgen and luteinizing hormone receptor (LHR) in granulosa and theca cells, as well as oocyte in response to hormonal stimulation and actions of intra-ovarian regulators [15]. MicroRNAs expression profile and analysis of their target genes revealed that microRNAs exhibits characteristics important in regulating downstream LHR signaling in ovarian cancer cells [41]. MiR-132, 5-fold down-regulation in DHT-treated group compared to control in this study, has previously been shown to be related to luteinizing hormones [12], raising the possibility that elevated LH in PCOS could involve the expression and action of miR-132.

Androgen Receptors (ARs) mRNA is predominantly expressed in rat granulosa cells and its expression decreases with the stage of follicle development [35]. Hyperandrogenemia induced by DHT is characterized by down-regulation of granulosa cell AR in rat preantral which could be restored by FSH administration [35]. Ovarian follicle expression of miR-221 and miR-222 has been reported to be repressed by androgens [42-44] which regulates cell proliferation by targeting p27/kip1 [44,45]. Here we observed higher expression of miR-222 in the DHT-induced PCOS rats, a response most evidenced in both theca and granulosa of early stage follicles. Interestingly, its expression pattern changes with increased follicular growth and antrum formation. In the cystic follicle, expression of miR-222 becomes restricted to theca only, suggesting that miR-222 modulates AR expression and hereby paracrine regulation.

Clinical studies have shown that follicular ERα and ERβ expression is significant altered in PCOS [46]. In breast cancer cells, over-expression of miR-221, miR-222, let-7 and miR-20b is associated with reduced of ERα protein content, signaling and expression of ERα target genes [47-49]. Our present study also showed higher expression of these miRNAs in the induced PCOS, consistent with a similar mechanism found in the breast cancer cells and their possible role in the regulation of ERα signaling. *In situ* localization shows specific expression of miR-222 in the theca cells (Figure 5) where it may be involved in regulation of the ERα. In addition to miR-221/222, several studies also highlighted the differential regulation of let-7d, let-7f, miR-25 and miR-26b in prostate and breast cancer, as well as in leukemia by the estrogen receptor pathways and that their expression was up-regulated in ERα-positive cells [50-52]. Expression analysis of these miRNAs revealed their higher expression in PCOS, raising the possibility that estrogen-mediated regulation of these miRNAs might be involved in the etiology of PCOS*.* Unlike other miRNAs in DHT-treated PCOS rats, miR-183 was found to be highly expressed in granulosa cells of control ovaries but was down-regulated by 10-fold in the PCOS counterpart. Interestingly, this miRNA was found to be correlated with estrogen receptor in breast cancer [53].

Hyperandrogenism in PCOS is one of the main causes for early follicle excess, which induces nuclear forkhead box transcription factors 3a (FOXO3a) exclusion and follicular arrest [54]. FOXO3a, a target of miR-96, promotes cancer cell proliferation [55]. Accordingly, expression of miR-96 in PCOS shows a 6-fold down-regulation compared to control ovary and is localized in the theca and cumulus-granulosa of cystic follicles. Thus, the cell proliferation in the preantral follicles in hyperandrogenic condition could be induced through a mechanism utilizing miR-96-mediated regulation.

PCOS is a complex syndrome with both reproductive and metabolic disorders. Although the rat PCOS model (DHT-treatment) simulates the hyperandrogenic state found in human PCOS and recapitulates some of the phenotypes of the human syndrome (obesity, disruption of menstrual cycle, insulin resistance), an inherent problem of any animal model for the complex syndrome is that it does not address all the phenotypes in PCOS. While the rat PCOS model may provide some mechanistic insight in the pathophysiology of the human syndrome, the significant findings from the animal studies need to be validated in the human context. In addition, although miRNAs are known to regulate multiple gene targets in different system [56,57], they can also be regulated by other paracrine/autocrine factors [44,58]. The precise target (s) for the reported dysregulated miRNA and their immediate down-stream processes need to be defined.

In summary, using an androgenized rat model which recapitulates many of the reproductive and metabolic phenotypes of human PCOS, we have shown that miRNAs are differentially regulated in a follicular cell-specific manner. Since one given miRNA could target multiple mRNAs of different genes and a given target may be regulated by multiple miRNAs [59], whether these miRNAs involve in the dysregulation of the hormone receptors (AR, FSHR and ER) and intra-ovarian regulators of ovarian follicle growth and function in PCOS is unknown. The expression of predicted target genes and their regulation by these miRNAs needs to be clarified by further experiments. Our ongoing studies not only include target gene expression analysis but also their correlation with the miRNAs, and functional analysis. Taken together, the present findings support the notion that the present DHT-treated rat model is useful for investigating the role and regulation of miRNAs in the molecular and cellular mechanism of PCOS and may offer new insights for the treatment of reproductive and metabolic disorders associated to PCOS.

## Abbreviations

PCOS: Polycystic ovarian syndrome; miRNA: microRNA; DHT: 5α-dihydrotestosterone; CL: Corpus luteum; CTL: Control; eCG: Equine chorionic gonadotropin; FC: Follicle cyst; AR: Androgen receptor; ER: Estrogen receptor; IST: Insulin sensitivity test; DIG: Digoxigenin; SSC: Sodium chloride/sodium citrate.

## Competing interests

The authors declare that they have no competing interests

## Authors’ contributions

MMH, MJC, QW and JYK performed the experiments, prepared the data and drafted the manuscript. KS, DT and BKT are co-mentors, provided input of studies and edited the manuscript. All authors read and approved the final manuscript.
